# Wet Etching of Quartz Using a Solution Based on Organic Solvents and Anhydrous Hydrofluoric Acid

**DOI:** 10.3390/ma15186475

**Published:** 2022-09-18

**Authors:** Yang Wan, Xinghe Luan, Longzao Zhou, Fengshun Wu

**Affiliations:** 1 School of Materials Science and Engineering, Huazhong University of Science and Technology, Wuhan 430074, China; 2 TKD Science and Technology Co., Ltd., Suizhou 441300, China

**Keywords:** wet etching, BOE solution, anhydrous etching solution, surface morphology, electrical properties

## Abstract

The quartz-crystal resonator is the core device for frequency control in modern communication systems and network technology. At present, in modern resonator blanks manufacturing, BOE solution is usually used as the etching solution, but its etching rate is relatively volatile, and the surface morphology of the blanks is prone to defects after etching, which brings certain difficulties to the deep-etching process of the wafer. To solve the above challenges, this paper systematically compares a BOE solution and anhydrous etching solution in terms of etching rate, surface morphology, and electrical properties of the blanks after etching. Seven groups of blanks were etched using different etching solutions with different etching conditions to verify their effect on the surface morphology and electrical properties of quartz blanks. The experimental results suggest that the application of anhydrous etching solution has achieved better surface morphology and electrical properties and can be more suitable for application in batch manufacturing. In general, when using anhydrous etching solution, it is possible to reduce surface roughness by up to 70% and equivalent resistance by 32%, and the etch rate is almost 10 times lower than BOE solution under the same temperature, which is more conducive to the rate control of wafers in the etching process.

## 1. Introduction

Quartz crystal has become one of the most widely used piezoelectric materials in the resonator manufacturing industry due to its unique piezoelectric properties and excellent mechanical properties [1]. Of all of the quartz crystal types, AT-cut quartz crystals are the most common type of resonator found due to their low processing costs and excellent thermal stability [2,3,4,5].

In modern resonator blanks manufacturing, some enterprises have used the wet etching process to replace the grinding and polishing process; they usually use NH_4_HF_2_ or BOE solution as an etching solution, but the surface of the wafer after etching is prone to wrinkles, twill, and other defects, which affect the electrical properties of the resonator blanks [6].

With the rapid advent of wireless communication technology and 5G applications, the market requires quartz resonators to have a smaller size, higher frequency, lower resistance, and better stability [7]. A lower equivalent resistance requires that the surface quality of the quartz wafer be increasingly higher. The main methods used to improve the surface quality of quartz wafers include lowering the etching temperature and adding surfactants to the etching solution [8,9,10,11,12]. However, there is little research on the effect of organic solution etching on the surface morphology and electrical properties of quartz wafers in wet etching. Recent research on quartz wet etching mainly focuses on the impact of the etching solution temperature and concentration on key characteristics of the resonator blanks such as the etching rate, the surface morphology of the wafer, and the equivalent electrical parameters of the quartz wafer [13].

In general, the etching process of quartz in HF acid can be expressed as:

$$S_{i}O_{2}+\mathrm{HF}\to S_{i}F_{4}+H_{2}O$$

In general, SiF_4_ is in the gaseous state, but it is not volatilized after production and will react further with HF acid:

$$S_{i}F_{4}+H_{2}O+\mathrm{HF}\to H_{2}S_{i}F_{6}+H_{2}S_{i}O_{3}$$

HF acid is a weak acid that is partially ionized in solution, i.e.,:

$$\mathrm{HF}↔H^{+}+F^{-}$$

The concentration of these particles in the solution follows the ionization equilibrium of the weak electrolyte [14,15,16]. The ionization equilibrium constants, *K*_1_ and *K*_2_, are temperature-dependent. At 25 °C, *K*_1_ = 6.85 × 10^−4^ mol/L and *K*_2_ = 3.963 mol/L. *K*_1_ and *K*_2_ can be expressed as follows:

$$K_{1}=c\left(H^{+}\right)\times c\left(F^{-}\right)/c\left(\mathrm{HF}\right)$$

$$K_{2}=c\left({\mathrm{HF}}_{2}^{-}\right)/\left(c\left(H^{+}\right)\times c\left(F^{-}\right)\right)$$

HF molecule can form (HF)_n_ molecule group through hydrogen bond. (HF)_2_ is one of the more common particles. The reaction to form (HF)_2_ is as follows:

$$\mathrm{HF}↔{\left(\mathrm{HF}\right)}_{2}$$

When the concentration of HF acid is greater than 1 mol/L, the (HF)_n_F^−^ ion is also synthesized, i.e.,:

$${\left(\mathrm{HF}\right)}_{n}+F^{-}↔{\left(\mathrm{HF}\right)}_{n}F^{-}$$

According to the quartz chemical-etching reaction process, there are H^+^, F^−^, HF_2_^−^, HF, (HF)_n_, and (HF)_n_F^−^ particles in HF acid solution [17].

Therefore, this paper compares the etching rate, surface morphology, and electrical parameter performance of wafers in BOE solution and anhydrous etching solution in order to guide the processing and manufacturing process of the resonator and improve the surface morphology and electrical properties of the quartz wafer.

## 2. Materials and Methods

### 2.1. Materials

**Figure 1:**
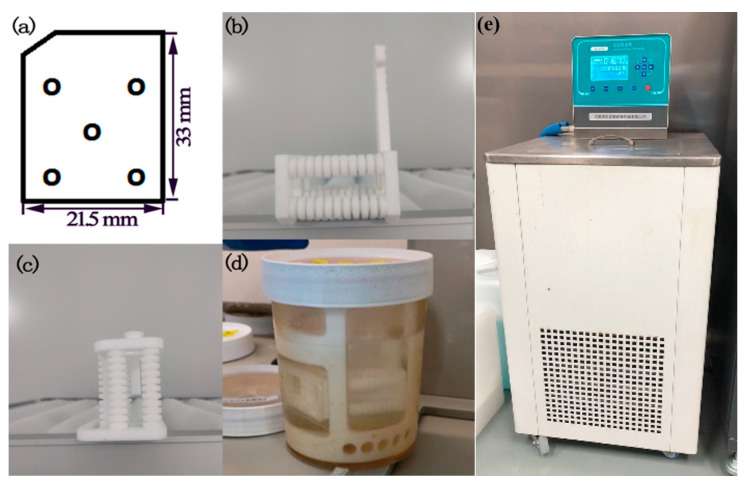
Etching wafers, fixtures, and equipment: (**a**) one-inch wafer and measurement points; (**b**) vertical jig; (**c**) horizontal jig; (**d**) etching tank; (**e**) thermostat tank.

The quartz blanks used in all of the tests as shown in Figure 1a have a length of 33 mm, a width of 21.5 mm, a thickness of about 0.042 mm and meet the conditions of Q value > 2.6 million, inclusions density class I, and etch-channel density ≤ 30 bars/cm^2^. The above quartz blanks were originated from “Stone Crystal Optoelectronics” in China and then processed by TKD Science and Technology Co., LTD (Suizhou, China) who cut, lapped, and polished the material. The initial roughness of the blanks selected in this round of tests were all in the range of 0.05–0.06 um. All blanks were cleaned with acid, alkali, and DI water before the experiments to ensure that the blank surfaces were free of particles and ionic contamination.

The aqueous etching solution used in this experiment is a BOE (buffered oxide etch) solution with a volume ratio of 1:6 (49% HF aqueous solution: 40% NH4F aqueous solution), and the anhydrous etching solution is made of dimethyl sulfoxide, surfactant, and anhydrous HF in a certain ratio (shown in Table 1). Some ingredients and concentrations have been omitted to protect trade secrets.

### 2.2. Equipment

The jig and etching tank, as shown in Figure 1b–d, used in this test were made of Teflon. The etching solution was poured into the etching tank before the test, and the etching tank was put into a precision constant temperature water bath for heating and insulation. The temperature-control accuracy of the bath was 0.05 °C. In order to ensure the accuracy of the temperature control during the etching process, a thermostat tank produced by Jiangsu Naile Instrument and Equipment Manufacturing Co., Ltd. (Nanjing, China), shown in Figure 1e, was used as the heating device.

**Figure 2:**
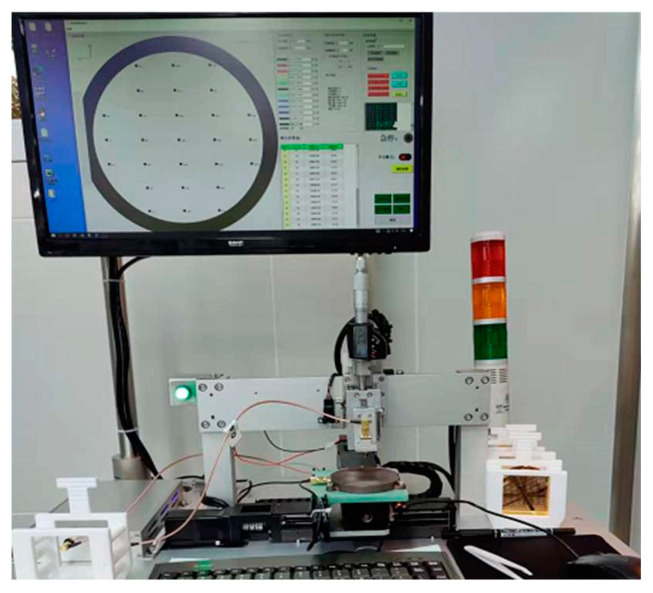
MT200 frequency tester.

This paper uses the MT200 frequency tester from Nanjing Automation Technology Co., Ltd. (Nanjing, China) (shown in Figure 2). The working principle of this model is that a digital frequency synthesizer generates a sine wave sweeping-frequency signal in the set frequency range, and the response signal is obtained after applying the signal to the upper and lower surfaces of the wafer. By comparing the amplitude of the input and output signals, the crystal impedance can be calculated, and the frequency at which the impedance suddenly changes is the resonant frequency of the quartz crystal.

### 2.3. Methods

The quartz blanks were divided into 7 groups and etched under different etching conditions. The etching conditions of each sample are shown in Table 2. To ensure the comparability of each group of wafers after etching, the etching depths of all wafers in this test are the same under different conditions. The determination of thickness of the quartz plates was made from frequency measurements. Thickness is be given in microns. Each data point was determined from the median value of 5 measurements (shown in Figure 1a). The accuracy of the measurement was estimated to be approximately 0.005 microns. An SPM9700 atomic-force microscope was used to measure the surface morphology of each group of wafers after etching. A 250 B network analyzer was used to measure the frequency and DLD scanning curve of each group of wafers after etching. W2220 crystal, a temperature–frequency hopping test system, was used to measure the temperature–frequency characteristic curve of each group of resonators.

## 3. Results

### 3.1. Temperature Factor

**Figure 3:**
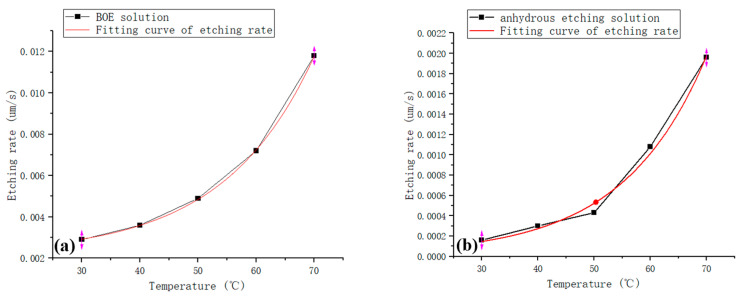
Temperature–etching rate relationship: (**a**) BOE solution; (**b**) anhydrous etching solution.

After the etching was completed, by measuring the frequency of the blanks of groups one and two, the etching rates of the above two etchants at different temperatures were calculated, and the effect of temperature on the etching rate was plotted, as shown in Figure 3.

From the above graph, we can see that the etching rates of BOE etchant and anhydrous etchant increase exponentially with an increase in temperature in the interval of 30~70 °C. Furthermore, the rate of anhydrous etchant is one order of magnitude smaller than that of BOE etchant, indicating that the rate stability of anhydrous etchant is higher in the same temperature fluctuation range, which is more conducive to the rate control of wafers in the etching process [17,18,19].

### 3.2. Water Content

**Figure 4:**
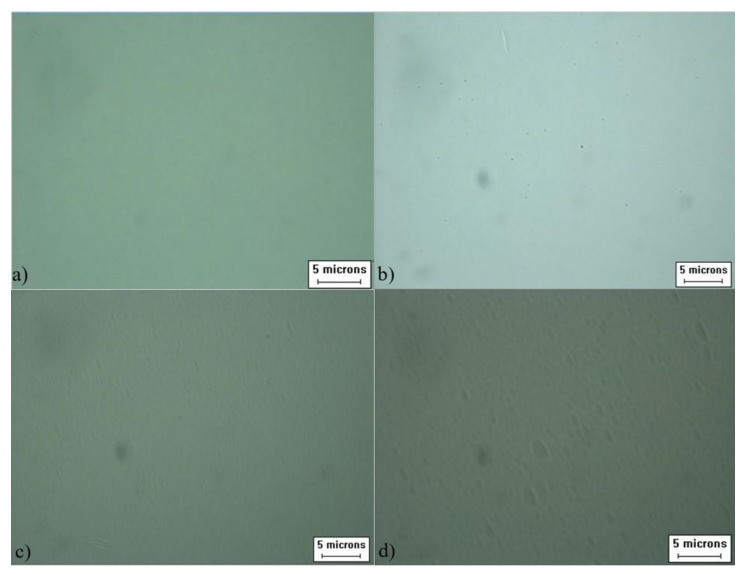
Comparison of the morphology of wafers etched in etching solutions with different water contents: (**a**) water-free; (**b**) the 3rd group; (**c**) the 4th group; (**d**) the 5th group.

**Figure 5:**
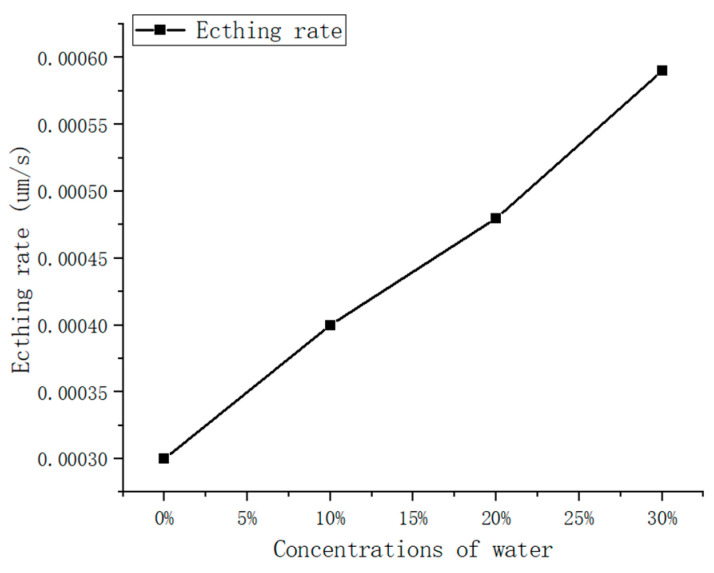
Etching rates under different water content conditions.

In order to analyze the effect of moisture content on the etching effect of anhydrous etching solution, the wafer surface morphologies of the third, fourth, and fifth groups of wafers were etched in the etching solution with different moisture contents at the same depth as shown in Figure 4, and the change in etching rate is shown in Figure 5.

As can be seen in Figure 4, the surface morphology of the wafer is good after etching in the etchant without water; after etching in the etchant with 10% volume ratio of water, a small amount of etch mound defects appear on the surface of the wafer; after etching in the etchant with 20% water, denser etch mound defects appear on the surface of the wafer; when the wafer is finished etching in the etchant with 30% water, the etch mound defects on its surface become denser and larger. In Figure 5, the calculated curves are shown to demonstrate the significance that the presence of water has on the etch rate.

### 3.3. Surface Morphology

**Figure 6:**
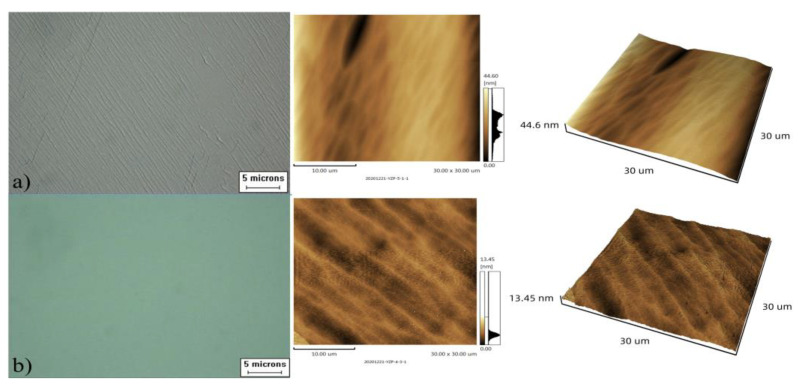
The morphology of each group after etching: (**a**) the 6th group; (**b**) the 7th group.

After the etching was completed, the etched surface morphology was observed by an optical microscope and an SPM9700 atomic-force microscope, respectively, and the results are shown in Figure 6. It can be seen from these figures that under different etching conditions, the surface of the wafer exhibits different surface features [20,21,22]. In the comparison in Table 3, the surface roughness of the wafer etched with anhydrous etching solution is lower than that of the wafer etched with BOE solution.

Between the two groups of wafers, the surface morphology of the seventh group of wafers observed by the optical microscope was mirror-like, without any defects such as twill or dark pits. Under the atomic-force microscope, the surface undulation and wrinkles were less, and the surface roughness was also the best.

### 3.4. Frequency Characteristics and Electrical Performance

**Figure 7:**
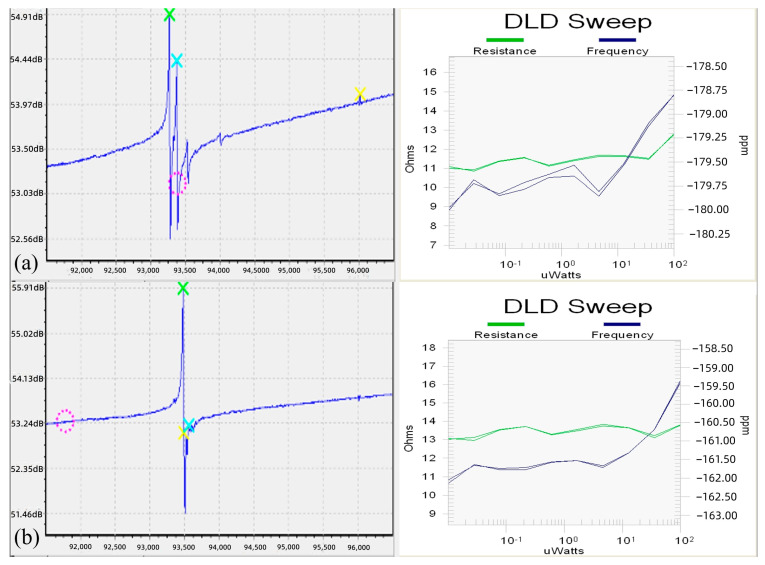
Frequency waveform and DLD scan curve after etching: (**a**) the 6th group; (**b**) the 7th group.

Figure 7 shows the frequency characteristics and DLD (drive-level dependence) of the blanks. As to frequency characteristics, by measuring the frequency waveforms of the two groups of blanks, it can be seen that the seventh group of blanks has the best frequency waveform without any parasitic waves. The parasitic wave in blanks of the sixth group become larger with higher roughness.

DLD characteristics are another important index of the resonator, as they can reflect the changes in resonator frequency and resistance under a different drive power [23,24,25]. Specifically, in the process of DLD testing, the higher the fitting degree of the two DLD curves, the better the stability of the resonator.

Under the same manufacturing process, these two groups of wafers were manufactured into resonators. The electrical performance parameters at room temperature of the seventh group of resonators are significantly better than the sixth group, as shown in Table 4.

### 3.5. Temperature–Frequency/Resistance Characteristics

**Figure 8:**
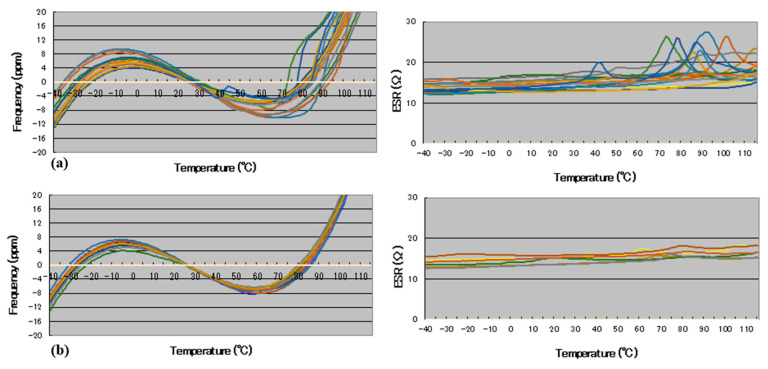
Temperature–frequency/resistance characteristic curves: (**a**) the 6th group; (**b**) the 7th group.

The temperature–frequency characteristic curve is an important indicator for evaluating the resonator because the resonator must have relatively stable performance under different environment conditions [26]. In this test, the frequency and the resistance of two groups of resonators were detected within the range from −40 °C to 105 °C at a detection interval of 2 °C. The results are shown in Figure 8.

It can be seen that the blanks in seventh group have the best stability, especially in the high-temperature region. This shows that the performance of the wafer is more stable with better surface morphology.

## 4. Discussion

The rate of dissolution of crystalline quartz in HF is thought to be an activation-controlled reaction, also referred to as “etching rate”. The basic formula for such a reaction and the basis for the investigation presented in this paper is as follows [27]:

$$Er=\frac{collisions}{second}\times probability\;factor\times energy\;factor$$

where

*Er* is the etching rate;
collisions is determined by the concentration of HF in the etch solution and the temperature of the etch [28,29]. These two variables will have an effect directly proportional to the number of collisions that will occur between the molecules in the crystalline quartz and the molecules in the acid solution;
probability factor is defined as the percentage of the number of collisions that will have sufficient energy and occur at the proper orientation to create a reaction;
energy factor is defined as the level of energy required to cause a reaction. The value is dependent on temperature only and is not affected by concentration or any other factors [30]. The value is represented by a well-established relationship first proposed by Arrhenius and forms the basis of an activation-controlled reaction. The result is exponential relative to temperature. The value is defined by the following equation [31]:
  $$energy\;factor=\exp\left(\frac{E_{a}}{R}\times T\right)$$
  where
  *E_a_* = activation energy (joules);
  *R* = universal gas constant;
  *T* = temperature (Kelvin).

Therefore, the basic equation for the rate of etching rate is:

$$Er=A\;\exp\left(\frac{E_{a}}{R}\times T\right)$$

The pre-exponential factor *A* is the product of the probability factor, concentration factor, and other modifiers [32].

For the BOE solution, a high proportion of ionization of HF leads to higher collisions and probability factor, which means that the etching process not only has a high rate, but also produces localized random etching, and deep etching tunnels will easily appear on the wafer surface. For anhydrous etching solution, HF can be linked to the organic solvent molecules through weak chemical bonds. It has the lower etching rate than that of the BOE solution at the same concentration of HF, but with better surface morphology. Lower etch rates mean easier control of the batch-etching process, and better surface topography means better electrical properties of the quartz blanks.

**Figure 9:**
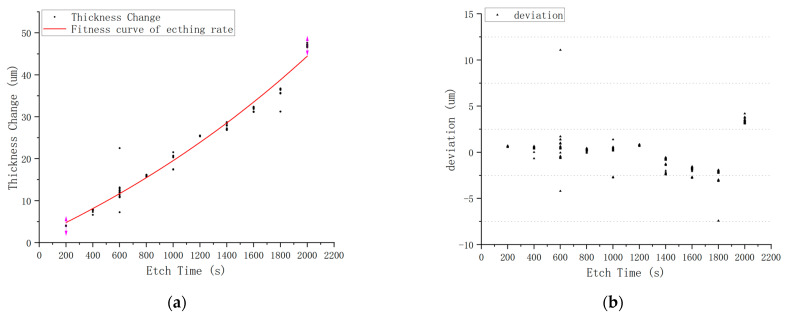
Etching rate of BOE solution at 60 °C: (**a**) etching rate; (**b**) deviation.

In Figure 9a, data taken from a production line are compared to a fitness curve. There are 2795 pieces of quartz blanks used to plot this graph and the etch solution was the BOE solution. In Figure 9b, the difference in the calculated curve vs. the actual data points is plotted.

**Figure 10:**
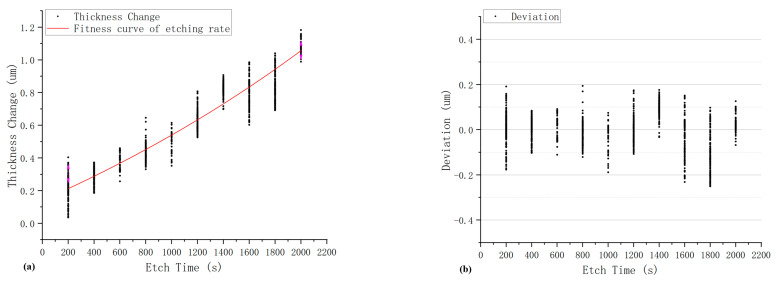
Etching rate of anhydrous etching solution at 60 °C: (**a**) etching rate; (**b**) deviation.

In Figure 10a, data are also taken from a production line and are compared to the fitness curve. There are 2992 pieces of quartz blanks used to plot this graph, and the etch solution was anhydrous etching solution. In Figure 10b, the difference in the calculated curve vs. the actual data points is plotted.

A comparison of Figure 9 and Figure 10 shows that at the same temperature and time condition, the etching rate in the anhydrous etching solution is much lower than BOE solution, and the deviation values range of anhydrous etching solution is from −0.23 um to +0.2 um, while that of BOE solution is from −7.5 um to +4.8 um, which means anhydrous etching solution is favorable for process control in the batch-etching process.

## 5. Conclusions

In this paper, by comparing the etching rate of blanks in different etching solutions, the surface morphology, and the electrical-property parameters after etching, the following conclusions are obtained:

1. Compared with the BOE solution, the anhydrous etching solution has a lower etching rate (nearly 10 times lower) and less fluctuation in the rate (almost 25 times smaller), which are good for process control in mass production.
2. For the anhydrous etching solution, it was found that even 10% of water content had a significant impact on the etch rate and the wafer surface quality.
3. The surface morphology and electrical-property parameters of quartz blanks etched in anhydrous etching solution are better than those etched in the BOE solution.
4. The quality of the surface morphology of the quartz wafer will directly affect the frequency waveform of the wafer and will have a significant impact on the electrical performance, DLD characteristics, and temperature–frequency characteristics of the resonator which is made by this wafer. The lower the surface roughness of the wafer, the better the above performance parameters will be.

In summary, it has been found by the author that etch solutions based on organic solvents and HF have many advantages over BOE solutions. They have a low etching rate, which means the etching process could be very stable and extremely predictable. Anhydrous etching solution enables quartz blanks to have good surface topography and excellent electrical properties after etching (including the frequency waveform, DLD characteristics, temperature–frequency characteristics, and temperature–resistance characteristics), which significantly improves the yield rate of quartz blanks during the manufacturing process.

## Figures and Tables

**Table 1:** Composition and concentration of BOE solution and anhydrous etching solution.

Solution	Parameter	Nominal	Limits	Measured
BOE solution	HF	25.6%	±0.5	25.49%
	NH_4_F	19.6%	±0.3	19.77%
	Particles (≥0.5 um, pcs/mL)	NA	≤160	33
	Hazen	NA	≤10	<10
	Chloride (Cl)	NA	≤3	<3
	Sulfate (SO_4_)	NA	≤1.5	<1.5
	Phosphate (PO_4_)	NA	≤0.5	<0.5
Anhydrous etching solution	HF Concentration	20.0 mol	±0.3	20.181 mol/L
	Sulfur Dioxide	NA	<15 ppm	0.327 ppm
	Fluorosilicic Acid	NA	<15 ppm	0.097 ppm
	Sulfuric Acid	NA	<25 ppm	0.64 ppm
	Water	NA	<0.03%	0.0075%
	Density (g/mL) at 25 °C	1.129	±0.005	1.129

**Table 2:** Etching conditions of each group.

Group	Etching Solution	Etching Temperature (°C)	Volume Ratio of Water and Solution
1	BOE solution	30/40/50/60/70	-
2	anhydrous etching solution	30/40/50/60/70	-
3	anhydrous etching solution	40	1:10
4	anhydrous etching solution	40	1:5
5	anhydrous etching solution	40	3:10
6	BOE solution	40	-
7	anhydrous etching solution	40	-

**Table 3:** Surface roughness comparison of each group after etching.

Group	Status	Ra (nm)	Rz (nm)	Rq (nm)
6	Before etching	0.545	41.547	3.869
7	Before etching	0.541	16.658	1.114
6	After etching	6.109	44.718	7.279
7	After etching	0.911	18.703	1.165

**Table 4:** Electrical performance parameters of the 6th and 7th groups of blanks at room temperature.

Group	R (Ω)	RLD2 (Ω)	DLD2 (Ω)	FDLD (PPM)	Quantity (pcs)
6	23.86	25.7	5.14	4.14	100
7	16.11	16.6	0.92	1.08	100

## Data Availability

Data are contained within the article.
